# Dietary vitamin K intake is associated with decreased neurofilament light chain among middle-aged and older adults from the NHANES

**DOI:** 10.3389/fnut.2024.1396707

**Published:** 2024-09-13

**Authors:** Jing Luo, Song Lin

**Affiliations:** ^1^School of Rehabilitation, Jiangsu College of Nursing, Huai'an, Jiangsu, China; ^2^Department of Clinical Nutrition, The Affiliated Huaian No. 1 People’s Hospital of Nanjing Medical University, Huai'an, Jiangsu, China

**Keywords:** vitamin K, neurofilament, neurodegenerative diseases, NHANES, middle-aged and older adults

## Abstract

**Purpose:**

Neurofilament-light chain (NfL) is associated with neurodegenerative diseases, which are increasingly prevalent with aging. Vitamin K has been shown a neuroprotective effect. Therefore, we aimed to explore the potential relationship between dietary vitamin K intake and serum NfL.

**Methods:**

This study was conducted on the 2013–2014 cycles of the National Health and Nutrition Examination Survey, a multi-site population-based study of the US general population. Serum NfL level was measured using a highly sensitive immunoassay. Dietary vitamin K intake was estimated from two-day dietary recall interviews, and its relationship with NfL was determined using linear regression models.

**Results:**

The study included a total of 1,533 participants with a median age of 46 years, comprising 801 women (52.2%) and 732 men (47.8%). The median dietary intake of vitamin K was 81.6 μg/d, and the median serum NfL was 12 pg./mL. After adjusting for potential confounding factors in the full model, individuals with higher dietary vitamin K intake had lower serum NfL levels (Q4 vs. Q1, *β* = −4.92, 95%CI: −7.66, −2.19, *p =* 0.002). A non-linear negative dose–response association is found between dietary vitamin K intake and serum NfL levels (*P* for non-linearity = 0.008); this association reaches a plateau when the dietary vitamin K intake is higher than 200 μg/d. According to the results of stratified analysis, the relationship between dietary vitamin K intake and serum NfL levels was stronger in the population of middle-aged and older adults.

**Conclusion:**

The present study suggested a negative association between dietary vitamin K intake and serum NfL levels in the general US population, especially in middle-aged and older adults. This study might offer a novel nutritional idea for the primary prevention and mechanism exploration of neurodegenerative diseases.

## 1 Introduction

Neurofilaments are constituted by intermediate filaments including heavy chain (NfH), medium chain (NfM), light chain (NfL) (1). These neurofilament proteins are synthesized in the neuronal perikaryon and integrated into the axonal cytoskeleton specifically (2). In normal neurons, the process of degradation of neurofilaments mainly depend on proteasomal and autophagocytic pathways (3). Neurofilament proteins can be released from neurons into cerebrospinal fluid (CSF) and blood after axonal injury (4). In neurofilament’s subunits, only NfL can self-assemble and constitute the backbone of nerve fibers (5). In addition, NfL has a small molecular weight (70 kDa) and high solubility, and has thus been extensively explored and proposed as a recognized biomarker of neurodegenerative diseases (6).

Early studies of NfL are based on CSF, while assays of NfL in peripheral blood are now available with the development of detection technology (7–9). Several epidemiological studies have proved a causal relationship between peripheral blood NfL and nervous system diseases (10–13). In the Chicago Health and Aging Project, a population-based cohort study, serum NfL levels are positively associated with the risk of stroke (12). Another longitudinal cohort study show that plasma NfL is independently associated with neurodegeneration in brain regions typically affected in Alzheimer Disease (13). In the Cardiovascular Health Study, its results show that circulating NfL is positive associated with risk of dementia and dementia-specific mortality (11).

Vitamin K have two biologically forms: K_1_ (phylloquinone) and K_2_ (menaquinone). Vitamin K1 is found in photosynthetic organisms. Vitamin K2 is derived from some obligate and facultative anaerobic bacteria and mainly found in dairy products (14). Vitamin K has been shown to be involved in brain health in recent years. Observational epidemiological studies have shown that dietary vitamin K is inversely associated with cognitive function (15–18). Menaquinone-4 is the main form of vitamin K in the brain (19). In the Rush Memory and Aging Project, its results show that higher brain menaquinone-4 concentrations are associated with a 17–20% lower odds of dementia or mild cognitive impairment (20). In a prospective cohort study, after 24 months of follow-up, geriatric patients using vitamin K antagonists show an obvious decline in executive function compared with their counterparts (21). These results suggest a potential protective effect of vitamin K on the nervous system. Vitamin K may exert its beneficial effects through several potential mechanisms, including the modulation of sphingolipid metabolism, anti-inflammatory properties, and protection against oxidative stress (22–26). These mechanisms are proposed to contribute to the preservation of neuronal integrity and function.

Although the roles of vitamin K and NfL in neural health have been studied individually, their relationship has not yet been thoroughly explored. Given the antioxidant and anti-inflammatory properties of vitamin K, which may contribute to the protective effects on neural cells (25), it is hypothesize that vitamin K may influence the release of NfL. However, to date, no epidemiological studies investigate the association between vitamin K and neurofilaments. Thus, the main aim of our study is to investigate whether dietary intakes of vitamin K are associated with serum NfL in the US general population.

## 2 Materials and methods

### 2.1 Study population

NHANES is an ongoing cross-sectional multistage, stratified program of the general US civilians. It is conducted by the National Center for Health Statistics to assess the health and nutritional status. To obtain a representative sample, non-Hispanic Asians, Hispanics, non-Hispanic Black, older adults, and low-income white individuals are oversampled. The details of the NHANES have been described in the previous study (27). NHANES program is approved by the Centers for Disease Control and Prevention Research Ethics Review Board (28). Documented signed consents are obtained from all adult individuals.

**Figure 1:**
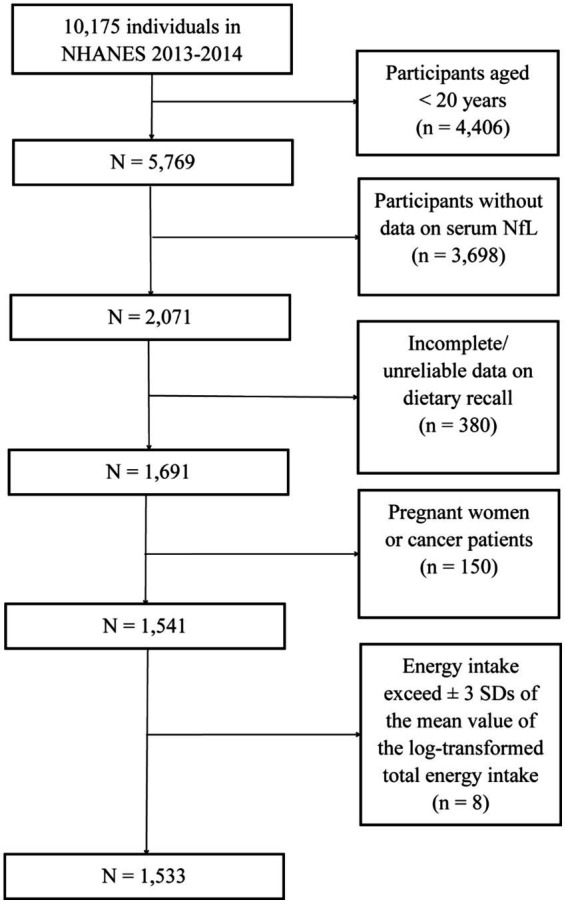
Flow chart of the study participants.

Because serum NfL was measured only in the 2013–2014 cycle of NHANES, this dataset was used for subsequent analyses. A total of 10,175 individuals participated in NHANES during 2013–2014. Among them, 5,769 individuals were 20 years of age and older. Participants without data on NfL (*n* = 3,698) and those with incomplete or unreliable dietary interview data (*n* = 380) were excluded. Additionally, pregnant individuals (*n* = 14), cancer patients (*n* = 136), and those whose energy intake exceeded ±3 SDs of the mean value of the log-transformed total energy intake (*n* = 8) were excluded, as these conditions could severely affect dietary intake. Finally, 1,533 participants with a median (IQR) age of 46 (33–59) years were included in the following analyses (Figure 1).

In our study, the regression model in this study included 14 variables. According to empirical rules, it was recommended that each predictor variable in a regression model should be supported by at least 20 observations to ensure reliable and stable estimates. Therefore, the minimum sample size required was 280. Our study included a total of 1,533 participants from the NHANES dataset, which significantly exceeded the required sample size of 280 participants. This large sample size provided ample power to detect meaningful relationships between the variables included in our model.

### 2.2 Serum neurofilament light chain

Blood samples were obtained via venipuncture and collected into blood collection tubes specifically designed to separate serum from other blood products. After collection, the serum was aliquoted into cryogenic vials and stored in a −70°C freezer according to test requirements. Participants aged 20–75 years in a half-sample from NHANES 2013–2014, who had stored surplus or pristine serum samples and consent to future research, were eligible.

In this study, a highly sensitive NfL immunoassay developed by Siemens Healthineers was utilized. This assay leveraged acridinium ester (AE) chemiluminescence and paramagnetic particles and was executed on the automated Atellica immunoassay system. The detailed procedure was as follows: initially, the sample was incubated with AE-labeled antibodies that specifically bound to the NfL antigen. After this binding phase, paramagnetic particles coated with capture antibodies were added to the sample, forming complexes with the AE-labeled antibody–antigen conjugates. Subsequently, the mixture underwent a separation process to remove any unbound AE-labeled antibodies. Following this, acid and base reagents were added to initiate the chemiluminescence reaction, and the resultant light emission was measured. The intensity of the emitted light correlated with the concentration of NfL in the sample.

The lower limit of quantification (LLOQ) of the NfL immunoassay was 3.9 pg./mL, which was determined by replicate testing of low-concentration samples (*n* = 44). The LLOQ was defined as the concentration at which the coefficient of variation (CV) was not more than 20%. The upper limit of quantification (ULOQ) was 500 pg./mL. For data below the LLOQ, an imputed value was equal to the LLOQ divided by the √2. For data above the ULOQ, an imputed value was equal to the ULOQ multiplied by the √2. Twenty-seven participants had values below the LLOQ. No participants had values above ULOQ.

### 2.3 Dietary intakes

All participants were eligible for two 24-h dietary recall interviews. The first dietary recall interview was collected in-person in the Mobile Examination Center (MEC) and the second interview was collected by telephone 3–10 days later.

The dietary intake data were used to estimate the types and amounts of foods and beverages consumed during the 24 h before the interview and to estimate intakes of energy, nutrients, and other food components according to the US Department of Agriculture Food and Nutrient Database (29). This database included comprehensive information that was used to code individual foods/beverages and portion sizes reported by participants and included nutrient values for calculating nutrient intakes. Approximately 10 percent of the coder’s work was randomly selected to be independently coded by another coder. Information regarding the consumption of dietary supplements was gathered through a 30-day dietary supplement survey. The mean quantity of vitamin K supplements consumed was ascertained by aggregating the total vitamin K supplements taken and then dividing this sum by 30. The total vitamin K consumption was estimated by combining the daily vitamin K intake from both dietary sources and supplementation.

### 2.4 Other variables

Participants’ self-reported information included age, sex, race, education, family income, smoking status, alcohol drinking status, and medical history. Alcohol drinkers were defined as those who consume alcohol at least 12 times per year. Smokers were defined as those who had smoked at least 100 cigarettes in their lifetime.

Standing height and weight were measured in the MEC by trained health technicians. Body mass index (BMI) was calculated as weight in kilograms divided by height in meters squared.

Diabetes mellitus was defined as participants who met one of the following items: fasting plasma glucose ≥126 mg/dL; hemoglobin A1c ≥ 6.5%; self-reported physician diagnosis of diabetes; or current taking anti-diabetic drugs (30). Hemoglobin A1c was measured using high-performance liquid chromatography, where hemoglobin components were separated based on ionic interactions, and their absorbance was measured at 415 nm to determine its percentage. Fasting glucose levels were determined using an enzymatic hexokinase method, in which glucose was phosphorylated to glucose-6-phosphate, and the resulting NADPH was quantified photometrically to measure glucose concentration. Detailed methods for measurements of fasting glucose and hemoglobin A1c were reported previously (31). The estimated glomerular filtration rate (eGFR) was calculated using the Chronic Kidney Disease Epidemiology Collaboration (CKD-EPI) equation (32).

Cardiovascular disease was defined as participants who had a self-reported physician diagnosis of either congestive heart failure, coronary heart disease, angina pectoris, heart attack, or stroke.

### 2.5 Statistical analysis

All analyses were performed using Stata version 15.1 (Stata Corporation, College Station, TX, United States). Considering the multi-stage design of NHANES, appropriate weight and sampling unit variables were designated according to the NHANES analytic guidelines. Qualitative variables were expressed by frequency distributions. Continuous variables were expressed by medians and inter-quartile range (IQR). General characteristics and serum NfL according to dietary vitamin K intake were evaluated using the chi-square test for categorical variables and linear regression for continuous variables. Linear regression models were fitted to assess the relationship between dietary vitamin K intake and serum NfL levels. Covariates were chosen based on literature knowledge. Age, sex, and race were adjusted for the first model. The second model was further adjusted for educational levels, BMI, smoking status, alcohol drinking status, family income, diabetes, and cardiovascular disease (CVD). Further adjusted were made for total daily energy intake according to the nutrient density model (33). The restricted cubic spline (RCS) model was applied to explore the potential dose–response relationship between dietary vitamin K intake and serum NfL levels. A two-tailed value of *p* < 0.05 is considered statistically significant.

## 3 Results

### 3.1 Baseline characteristics across quartiles of vitamin K

**Figure 2:**
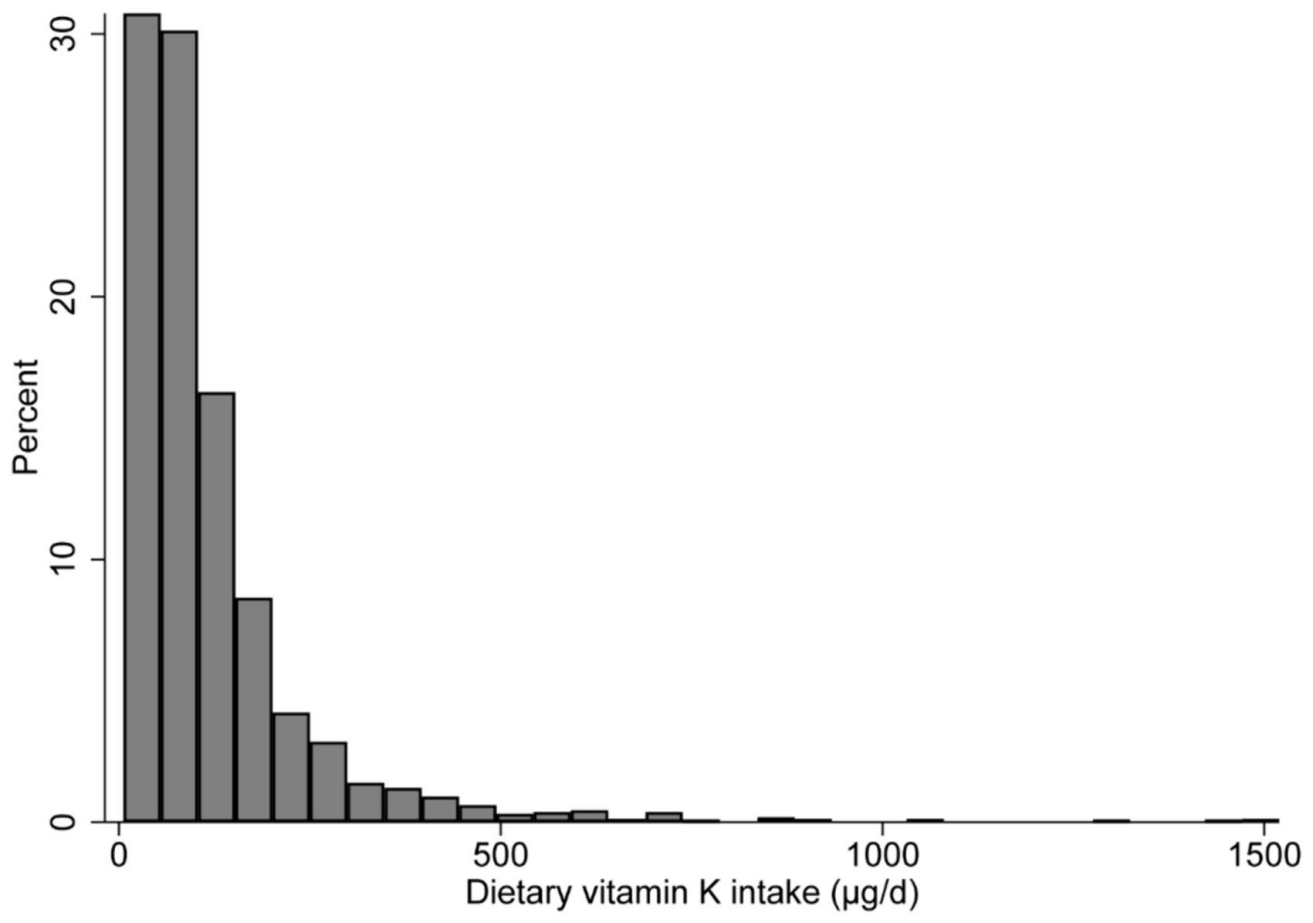
Distribution of dietary vitamin K intake.

The histogram shows that dietary vitamin K distribution is non-normal (Figure 2). The median (IQR) dietary intake of vitamin K is 81.6 (47.4–142.8) μg/d, and the median (IQR) serum NfL is 12.0 (8.0–18.5) pg./ml. Compared with participants in the lowest quintile of vitamin K intake, those in the highest quartile are more likely to have a higher degree of education, family income, BMI, dietary energy intake, and serum NfL levels; while those in the highest quintile are less likely to be Non-Hispanic White individuals, smokers, and CVD patients (Table 1).

**Table 1:** Characteristics of study population.

	Dietary vitamin K quartiles, μg/d				*P*
	Q1 (5.8–47.7)	Q2 (47.8–81.6)	Q3 (81.7–142.2)	Q4 (≥142.3)	
Number of subjects	384 (25.0)	384 (25.0)	382 (25.0)	383 (25.0)	
Age, years					0.441
20–45	198 (51.6)	180 (46.9)	179 (46.9)	193 (50.4)	
46–75	186 (48.4)	204 (53.1)	203 (53.1)	190 (49.6)	
Gender					0.978
Male	180 (46.9)	186 (48.4)	183 (47.9)	183 (47.8)	
Female	204 (53.1)	198 (51.6)	199 (52.1)	200 (52.2)	
Race/Ethnicity					<0.001
Mexican American	44 (11.4)	65 (16.9)	63 (16.5)	51 (13.3)	
Other Hispanics	31 (8.1)	46 (12.0)	37 (9.7)	36 (9.4)	
Non-Hispanic White individuals	182 (47.4)	165 (43.0)	163 (42.7)	150 (39.2)	
Non-Hispanic Black	86 (22.4)	69 (18.0)	63 (16.5)	63 (16.4)	
Other Race	41 (10.7)	39 (10.1)	56 (14.6)	83 (21.7)	
Education					<0.001
<High school	114 (29.7)	92 (24.0)	69 (18.1)	43 (11.3)	
High school	97 (25.3)	82 (21.3)	82 (21.5)	56 (14.7)	
>High school	173 (45.0)	210 (54.7)	230 (60.4)	282 (74.0)	
Family income, $					<0.001
<55,000	264 (71.5)	229 (61.9)	199 (54.4)	164 (43.9)	
≥55,000	105 (28.5)	141 (38.1)	167 (45.6)	210 (56.1)	
BMI, kg/m^2^					0.026
<25	104 (27.5)	109 (28.5)	93 (24.5)	129 (33.9)	
25 to <30	124 (32.7)	116 (30.3)	118 (31.0)	128 (33.6)	
≥30	151 (39.8)	158 (41.2)	169 (44.5)	124 (32.5)	
Alcohol drinker	271 (73.8)	269 (73.7)	273 (75.0)	276 (74.2)	0.979
Smoker	197 (51.3)	172 (44.8)	146 (38.2)	140 (36.5)	<0.001
Diabetes	56(14.6)	60 (15.6)	64 (16.8)	43 (11.2)	0.154
CVD	30 (7.8)	39 (10.2)	25 (6.5)	17 (4.4)	0.02
eGFR, mL/min/1.73m^2^	97.2 (81.4, 111.3)	96.9 (82.8, 109.7)	95.0 (80.3, 109.2)	96.5 (82.3, 109.5)	0.658
Serum NfL, pg./ml	12.5 (8.1, 20.0)	12.6 (8.7, 18.8)	11.6 (7.5, 18.4)	11.1 (8.1, 16.8)	0.008
Energy intake, kcal/d	1568.5 (1190.7, 1983.7)	2029.0 (1627.7, 2484.3)	2134.5 (1645.5, 2698.5)	2168.0 (1676.0, 2671.0)	<0.001

Data expressed as median [IQR] or *n* (%), unless otherwise stated.BMI, indicates body mass index; CVD, cardiovascular diseases; eGFR, estimated glomerular filtration rate; NfL, neurofilament light chain.

### 3.2 Associations between vitamin K intake and serum NfL levels

The results show an inverse association between dietary vitamin K intake and serum NfL (Table 2). In the age, sex, and race adjusted model (model 1), compared with participants in quartile 1, dietary vitamin K intake in quintile 4 is negatively associated with serum NfL levels (*β* = −4.58, 95%CI: −6.34, −2.80, *p* < 0.001). Associations between dietary vitamin K intake and serum NfL levels still exist after adjusting for potential confounders (model 2), including dietary energy intake (model 3).

**Table 2:** Association of dietary vitamin K intake with serum neurofilament light chain levels.

	Model 1	*P*	Model 2	*P*	Model 3	*P*
Quartile 1	Ref.		Ref.		Ref.	
Quartile 2	−2.24 (−5.29, 0.80)	0.137	−2.85 (−5.81, 0.10)	0.057	−3.12 (−6.69, 0.44)	0.081
Quartile 3	−1.34 (−6.07, 3.38)	0.554	−1.57 (−6.39, 3.24)	0.496	−1.91 (−6.78, 2.96)	0.416
Quartile 4	−4.58 (−6.34, −2.80)	<0.001	−4.58 (−6.53, −2.62)	<0.001	−4.92 (−7.66, −2.19)	0.002

Model 1 adjusted for age, gender, and race; model 2 adjusted for all covariates in model 1 plus educational levels, body mass index, smoking status, alcohol drinking status, family income, diabetes, and cardiovascular diseases; model 3 adjusted for all covariates in model 2 plus total energy intake.

### 3.3 Dose–response relationship

**Figure 3:**
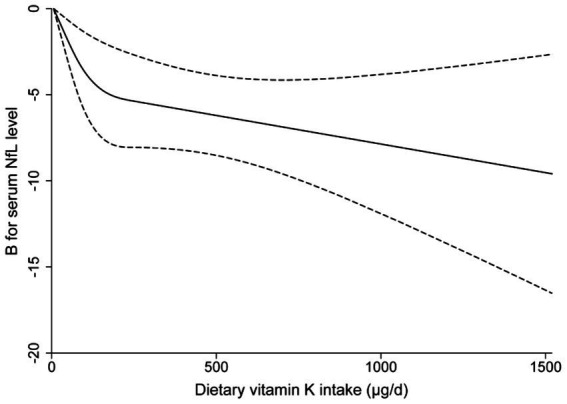
Multivariable-adjusted restricted cubic spline curve for the association between dietary vitamin K intake and serum neurofilament light chain levels. The solid line represents the fitting curve; the dashed line represents the confidence interval.

The RCS analysis indicate evidence against linearity (*P* for non-linearity = 0.008). When dietary vitamin K intake is below 200 μg/d, the serum NfL levels decrease rapidly with the increase of dietary vitamin K intake; while this association reach a plateau when the dietary vitamin K intake is higher than 200 μg/d (Figure 3).

### 3.4 Stratified analyses

The association between dietary vitamin K intake and serum NfL levels is modified by CVD (*P* for interaction = 0.042). No significant interaction is found between dietary vitamin K intake and other covariates, including age, sex, race, educational levels, BMI, smoking status, alcohol drinking status, family income, and diabetes (Table 3). Stratified analyses show a significant negative relationship between dietary vitamin K intake and serum NfL levels across each subgroup of educational levels, BMI categories, smoking status, alcohol drinking status, family income, and diabetes. These associations are enhanced in some subgroups (middle-aged and older adults, obesity, who have diabetes or CVD) (Table 3).

**Table 3:** Association of dietary vitamin K intake with serum neurofilament light chain levels stratified by covariates.

		Quartile 1	Quartile 2	*P*	Quartile 3	*P*	Quartile 4	*P*	*P* for interaction
Age	20–45	Ref.	−2.75 (−6.78, 1.28)	0.166	−2.97 (−6.69, 0.76)	0.11	−2.53 (−5.15, 0.08)	0.056	0.198
	46–75	Ref.	−1.20 (−5.99, 3.59)	0.602	0.75 (−7.03, 8.54)	0.84	−5.86 (−10.27, −1.46)	0.012	
Gender	Male	Ref.	−2.51 (−7.57, 2.54)	0.307	0.81 (−8.90, 10.53)	0.861	−4.55 (−9.76, 0.65)	0.082	0.289
	Female	Ref.	−3.48 (−8.44, 1.49)	0.157	−4.51 (−9.38, 0.36)	0.067	−5.05 (−8.69, −1.40)	0.01	
Race/ethnicity	Non-Hispanic White individuals	Ref.	−3.36 (−8.03, 1.32)	0.147	−2.98 (−8.94, 2.97)	0.302	−5.56 (−8.81, −2.30)	0.002	0.44
	Other race	Ref.	−2.91 (−7.19, 1.36)	0.167	−0.51 (−5.24, 4.21)	0.82	−4.91 (−11.29, 1.48)	0.122	
Education	<High school	Ref.	−1.81 (−7.71, 4.08)	0.522	−1.35 (−6.72, 4.03)	0.601	−4.61 (−8.92, −0.29)	0.038	0.549
	≥High school	Ref.	−3.25 (−7.84, 1.35)	0.153	−1.84 (−7.98, 4.29)	0.531	−4.92 (−8.37, −1.46)	0.008	
Family income	<55,000	Ref.	−1.79 (−6.16, 2.57)	0.396	2.95 (−4.60, 9.10)	0.495	−3.81 (−7.13, −0.47)	0.028	0.84
	≥55,000	Ref.	−4.91 (−10.96, 1.14)	0.104	−5.98 (−10.99, −0.97)	0.022	−6.48 (−11.19, −1.77)	0.01	
BMI	<30	Ref.	−2.84 (−6.71, 1.04)	0.14	−1.49 (−8.18, 5.20)	0.643	−3.18 (−5.57, −0.78)	0.013	0.264
	≥30	Ref.	−3.64 (−9.27, 1.98)	0.188	−4.08 (−10.90, 2.73)	0.221	−8.49 (−15.15, −1.84)	0.016	
Smoking	Smoker	Ref.	−1.87 (−7.22, 3.47)	0.467	0.63 (−7.45, 8.71)	0.871	−4.34 (−7.48, −1.20)	0.01	0.423
	Non-smoker	Ref.	−3.81 (−7.62, −0.10)	0.049	−3.91 (−8.81, 0.99)	0.110	−5.64 (−9.29, −2.00)	0.005	
Alcohol drinking	Drinker	Ref.	−1.42 (−4.33, 1.48)	0.312	−0.05 (−5.21, 5.10)	0.982	−2.38 (−4.26, −0.49)	0.017	0.064
	Non-drinker	Ref.	−10.20 (−18.30, −2.11)	0.017	−7.78 (−19.20, 3.65)	0.167	−14.26 (−23.53, −5.00)	0.005	
Diabetes	Yes	Ref.	−11.08 (−27.60, 3.99)	0.132	−8.18 (−21.97, 5.60)	0.225	−23.18 (−41.45, −4.90)	0.016	0.22
	No	Ref.	−1.69 (−4.77, 1.39)	0.261	−0.96 (−5.79, 3.86)	0.677	−3.14 (−5.35, −0.93)	0.008	
CVD	Yes	Ref.	−5.91 (−13.90, 2.87)	0.145	−6.91 (−15.90, 2.08)	0.13	−9.48 (−19.08, 0.13)	0.053	0.042
	No	Ref.	−3.48 (−7.19, 0.24)	0.065	−1.78 (−7.16, 3.59)	0.49	−4.65 (−7.44, −1.86)	0.003	

BMI, body mass index; CVD, cardiovascular diseases; NfL, neurofilament light chain.

### 3.5 Sensitivity analyses

Given the influence of the digestive system on dietary intake, participants who have liver, stomach, or intestinal diseases are excluded (*n* = 162). Compared with participants in quartile 1, dietary vitamin K intake in quintile 4 is negatively associated with serum NfL levels (*β* = −4.58, 95%CI: −7.19, −1.96, *p =* 0.002). In addition, after adjusting for fat, fiber intake, and eGFR, higher dietary vitamin K intake is also correlated with lower serum NfL levels (*β* = −4.59, 95%CI: −7.88, −1.30, *p =* 0.009). A total of 339 participants report that they are taking vitamin K supplements. The intake of vitamin K from both diet and supplements is integrated for a thorough analysis. The median (IQR) total intake of vitamin K is 82.0 (47.9–142.8) μg/d. The finding reveal that the correlation between total vitamin K intake and serum NfL levels persists, albeit with a minor variation (Q4 versus Q1, *β* = −5.03, 95% CI: −7.76, −2.31, *p* = 0.001).

## 4 Discussion

This large-scale, representative survey of US individuals reveals a novel inverse association between dietary vitamin K intake and serum NfL levels. Our dose–response analysis indicates that this relationship plateaus when dietary vitamin K intake exceeds 200 μg/d. Furthermore, the association appears to be more pronounced in middle-aged and older adults. These findings suggest that higher vitamin K intake may potentially mitigate neurodegenerative processes, as evidenced by lower serum NfL levels. Clinically, this could imply that increasing dietary vitamin K might be a viable strategy for neuroprotection, particularly in aging populations.

According to the latest American dietary guidelines, the recommended adequate intake (AI) of vitamin K is 90 μg/d for women and 120 μg/d for men, respectively (34). In the present study, the median dietary intake of vitamin K is 81.6 μg/d, only 594 (38.7%) participants comply with the AI standards of vitamin K; among them, 227 (31.0%) men and 367 (45.8%) women meet the standards. Similarly, in another NHANES from 1999 to 2010 (30,899 older adult, mean age 46.9 years), the mean dietary intake of vitamin K is 89.7 μg/d (35). Although acute severe illness is uncommon under the current amount of vitamin K intake, its long-term insufficient intake may influence the activation of vitamin K-dependent proteins (e.g., growth-arrest specific 6 and protein S) and sphingolipids synthesis in nervous system (22, 23, 26). Growth-arrest specific 6 and protein S demonstrate several neuron-protective effects, including promoting proliferation, anti-apoptotic, and anti-inflammation activity, thereby reducing neuronal damage and consequently lowering the release of NfL (36). Sphingolipids are an essential component of neuronal and myelin membranes, and its metabolic disorder may damage neuronal cell membranes and increase the release of NfL, and associated with neurodegenerative diseases (24). Vitamin K exhibits anti-inflammatory and antioxidant effects, capable of mitigating the harm to neural cells inflicted by oxidative stress and inflammatory mediators, thus may contributing to a reduction in NfL levels (25).

Although there is no study to explore the relationship between dietary vitamin K intake and serum NfL levels prior to our study, several studies suggest a potential neuroprotective effect of vitamin K. The NHANES (2,524 older adult, age ≥ 60 years) from 2011 to 2014 show that, compared with the lowest dietary vitamin K intake group (≤47.5 μg/d), the highest quartile of dietary vitamin K intake (>136.6 μg/d) is inversely related with cognitive performance (37). The PREDIMED-Plus study (5,533 older adult Mediterranean population, 48.1% women, age 65.1 ± 4.9 years with overweight/obesity and metabolic syndrome) report that, compared with a decrease in the intake of vitamin K (median − 97.8 μg/d), the highest tertile of 2-year change of dietary vitamin K intake (median 194.4 μg/d) is inversely associated with cognitive function scores (16). In an animal study conducted by Chatterjee et al. (38), menaquinone can protect against from aluminum chloride-mediated cognitive decline via reducing oxidative stress, inflammation, and β-amyloid deposition. Another animal study show that menaquinone can protect against intestinal dysbiosis associated hippocampus neuronal damage (39). These epidemiological and animal studies are mainly focused on cognitive impairment under senility and pathological states. In our study, although most subgroup analyses support the overall results, associations between vitamin K intake and serum NfL levels are magnified in subpopulations with older age, higher BMI, diabetes, and CVD. Further experimental and epidemiological studies are needed to explore this heterogeneity arising from senescence and metabolic disorder.

Interestingly, in the present study, the RCS analysis suggest a saturation effect in the association between vitamin K intake and serum NfL levels: when dietary vitamin K intake exceed 200 μg/d, the strength of its association increase slowly. Whether this phenomenon is related to vitamin K receptors on neurons or other potential mechanisms remain to be revealed. A vitamin K intake of 200 μg/d is considered safe because multiple randomized controlled trials show no adverse events with vitamin K supplementation (at least 12-week) above this dose (40–42).

Our study has several strengths. First, the results are based on large representative samples which are randomly drawn from multi-center, multi-ethnic, apparently healthy, and cover a wide age range, thus our findings can be extrapolated to the general population. Second, our findings are robust. Normal gastrointestinal and hepatic functions are essential for the absorption and utilization of vitamin K. After adjusting potential confounding factors, the association between vitamin K intake and serum NfL levels change little when participants with liver, stomach, or intestinal diseases are excluded. Third, based on the potential dose–response relationship identified in this study, researchers and clinicians can further explore the neuroprotective thresholds of vitamin K and conduct relevant clinical intervention studies. Finally, stratified analyses of our study provide clues to identify potential high-risk individuals who may benefit from a diet rich in vitamin K.

At the same time, our study has several limitations. First, this study is the cross-sectional design, thus the direction of causality cannot be ascertained and the residual confounding cannot be completed excluded. Second, a potential limitation of this study is that the observed associations between NfL levels and vitamin K intake may be influenced by the inherent variability in dietary measurements, which could affect the precision of our findings. However, this does not diminish the overall trend observed, and further research with more controlled dietary assessments is warranted to confirm these results. Thirdly, a notable limitation is the age of the participants, which may limit the applicability of the findings to older individuals who are more affected by neurodegenerative diseases. Finally, dietary intake levels should not be equated with levels in blood, CSF, and nerve cells, because dietary vitamin K can be transformed to the corresponding metabolite products.

## 5 Conclusion

In conclusion, our results indicate that dietary vitamin K intake is negatively associated with serum NfL levels among US general population, especially among middle-aged and older adults. This finding require confirmation in well-designed prospective cohort study or clinical trial in which vitamin K exposure is measured from three dimensions: dietary intake, blood, and nervous system. Additionally, mechanistic studies are essential to elucidate the underlying mechanisms of action and to better understand how vitamin K influences neurodegenerative processes.

## Data Availability

The data analyzed in this study are publicly available and can be accessed at: https://www.cdc.gov/nchs/nhanes/.
